# *In vitro* 50 Hz magnetic field long-term exposure: Cytogenetic tests on human lymphoblastoid TK6 cells and validation of the test environment

**DOI:** 10.1016/j.mex.2020.101071

**Published:** 2020-09-26

**Authors:** Ha Nguyen, Maryse Ledent, Véronique Beauvois, Roel Anthonissen, Luc Verschaeve, Jean-Francois Collard, Maurice Hinsenkamp, Veronique Feipel, Birgit Mertens

**Affiliations:** a Scientific Direction Chemical and Physical Health Risks, Sciensano, Belgium; b Universite Libre de Bruxelles, Belgium; c Universite de Liege, Belgium

**Keywords:** Micronucleus test, Comet assay, Extremely low-frequency magnetic fields, Background magnetic field, Shielding system

## Abstract

Potential health effects of extremely low-frequency (electro)magnetic fields (ELF-(E)MFs) have long been investigated, but the results are still inconclusive. With respect to genotoxicity, sound data related to the effects of long-term exposure to ELF-(E)MFs on the genetic material and the impact of long-term pre-exposure to ELF-(E)MFs on the sensitivity of cells to the damage induced by known mutagens are needed. In this manuscript, an optimized protocol for a combined *in vitro* comet/micronucleus study to investigate these effects in a human lymphoblastoid cell line (TK6) is provided including the description of a well-validated exposure system. Furthermore, the use of a shielding system to limit background ELF-MFs inside the incubator is described as well.

• Optimized protocols for cytogenetic tests with ELF-MFs on TK6 cells ensure the reproducibility of test results.
• Validation of exposure environment and exposure system are needed prior to performing tests with ELF-MFs.
• A simple, but effective method to shield cells and reduce unintentional ELF-MF exposure consists of using the mu-metal cylinder. This is of particular interest when studying the effects of low exposure levels.

## Graphical abstract

**Figure:**
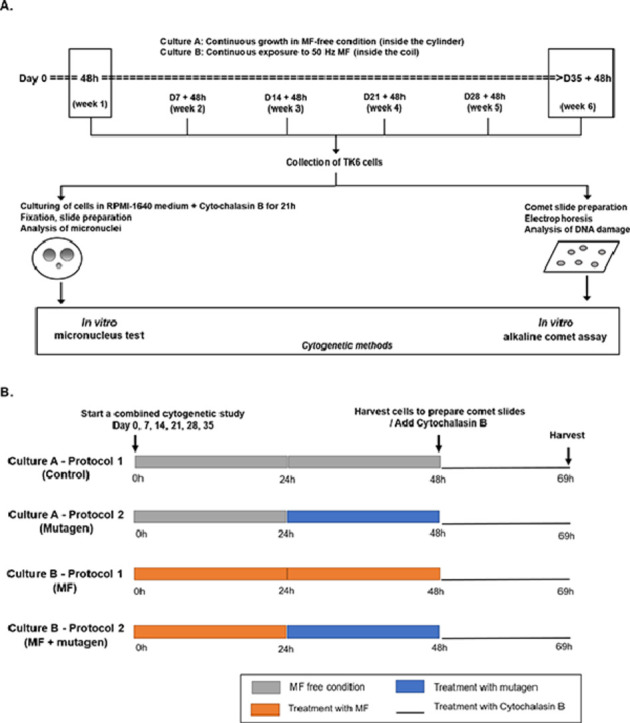

Specifications Table

**Table:** 

Subject Area:	Pharmacology, Toxicology and Pharmaceutical Science
More specific subject area:	Cytogenetic tests, non-ionizing radiation exposure
Method name:	*In vitro* comet assay, *in vitro* micronucleus test
Name and reference of original method:	*In vitro* comet assay • Collins, A. R., Oscoz, A. A., Brunborg, G., Gaivao, I., Giovannelli, L., Kruszewski, M., Smith, C.C & Štětina, R. (2008). The comet assay: topical issues. *Mutagenesis*, 23(3), 143-151 [1]. • Tice, R. R., Agurell, E., Anderson, D., Burlinson, B., Hartmann, A., Kobayashi, H., Miyamae, Y., Rojas, E., Ryu, J.C & Sasaki, Y. F. (2000). Single cell gel/comet assay: guidelines for in vitro and in vivo genetic toxicology testing. *Environmental and molecular mutagenesis*, 35(3), 206-221 [23].
	*In vitro* micronucleus test • Fenech, M. (2007). Cytokinesis-block micronucleus cytome assay. *Nature protocols*, 2(5), 1084 [4]. • OECD. 2014. OECD TG487: In vitro Mammalian Cell Micronucleus Test [18].
Resource availability:	• Fluorescent microscope (AxioImager.Z2) supplied with a camera and connected to Automated Scanning System • Mu-metal cylinder (Meca Magnetic, France)

## Background

Despite much research worldwide, no firm conclusion on the potential health effects of exposure to non-ionizing extremely low-frequency (electro)magnetic fields (ELF-(E)MFs) (1-300 Hz) can be made. While recent epidemiological studies still point towards an association between childhood leukemia and 50 Hz magnetic fields (MF), straightforward experimental data to support a causal relationship are lacking [3,^11^,^13^,^14^,21]. For this reason, *in vitro* and *in vivo* studies using improved methodologies have been set up to test existing as well as new innovative hypotheses on the possible effects of ELF-(E)MFs. However, results remain inconclusive, highlighting the need for replication of studies using sound experimental protocols [21].

To improve the reliability and reproducibility of *in vitro* studies to evaluate the impact of ELF-(E)MF exposure on the genetic material, a thoroughly validated exposure system as well as carefully designed exposure and testing protocols of the cytogenetic assays are of utmost importance. So far, *in vitro* cytogenetic studies towards the long-term effects of ELF-(E)MFs are less common [6], and a consistent methodology is lacking. In the first part of this manuscript, an optimized protocol for a combined *in vitro* cytogenetic study (i.e. the *in vitro* comet assay (COM) and the *in vitro* micronucleus test (MN)) in the human lymphoblastoid cell line (TK6) to investigate the effects associated with long-term ELF-MF exposure is provided. Moreover, by including some additional experimental conditions, the protocol can also be applied to examine the impact of long-term pre-exposure to ELF-MFs on the sensitivity of cells to damage induced by known chemical mutagens. Focus is deliberately put on the long-term exposure to 50 Hz MF, which is the frequency of the power network in Europe. Previous studies of our research group showed that cells pre-exposed for short-term to a low level of 50 Hz MF were less sensitive to chemical mutagens (i.e. showed less DNA damage) compared to cells exposed to the mutagen only [14]. Other similar examples have been reported in the literature [2,24], and therefore, this phenomenon requires further investigation.

The second part of the manuscript is dedicated to precautions that should be taken to ensure a high quality exposure system and test environment. Both measures to control ELF-MF levels in exposed cells as well as to reduce background ELF-MF exposure are discussed. The latter might contribute to unintentional ELF-MF exposure of the control cells. Although the presence of ELF-MFs inside an incubator has been stressed by many researchers [9,^17^,^19^,20], to our knowledge, there are only a few studies that take this phenomenon into account in their experimental design. As the ambient MF inside an incubator which has not been modified for MF bioeffect studies can range up to a few tenths µT [9], minimizing the MF exposure levels of the control cells is crucial, especially when studying low intentional exposure levels such as 10 or 50 µT. For this reason, precautions to ensure adequate exposure conditions are presented.

*Note:* The magnetic field strength is expressed in Amperes per meter (A/m) and the magnetic flux density, considering the magnetic permeability of a material is expressed in Tesla (T or µT). Since the term Magnetic Field (MF) is widely used in the literature, this is the terminology we use here, expressed in µT.

## Protocol for combined *in vitro* cytogenetic study on long-term effects of ELF-MF exposure

### General protocol description

The COM and MN are well-established tests that have been commonly used to investigate the potential effects of ELF-(E)MF exposure on the genetic material. Although in general these techniques are applied separately, they can also be combined to get a more complete picture of the genotoxic potential of ELF-(E)MF [10,24]. To our knowledge, no combined COM-MN study on the effects of long-term exposure to ELF-MFs in TK6 cells has been published yet.

**Fig. 1:**
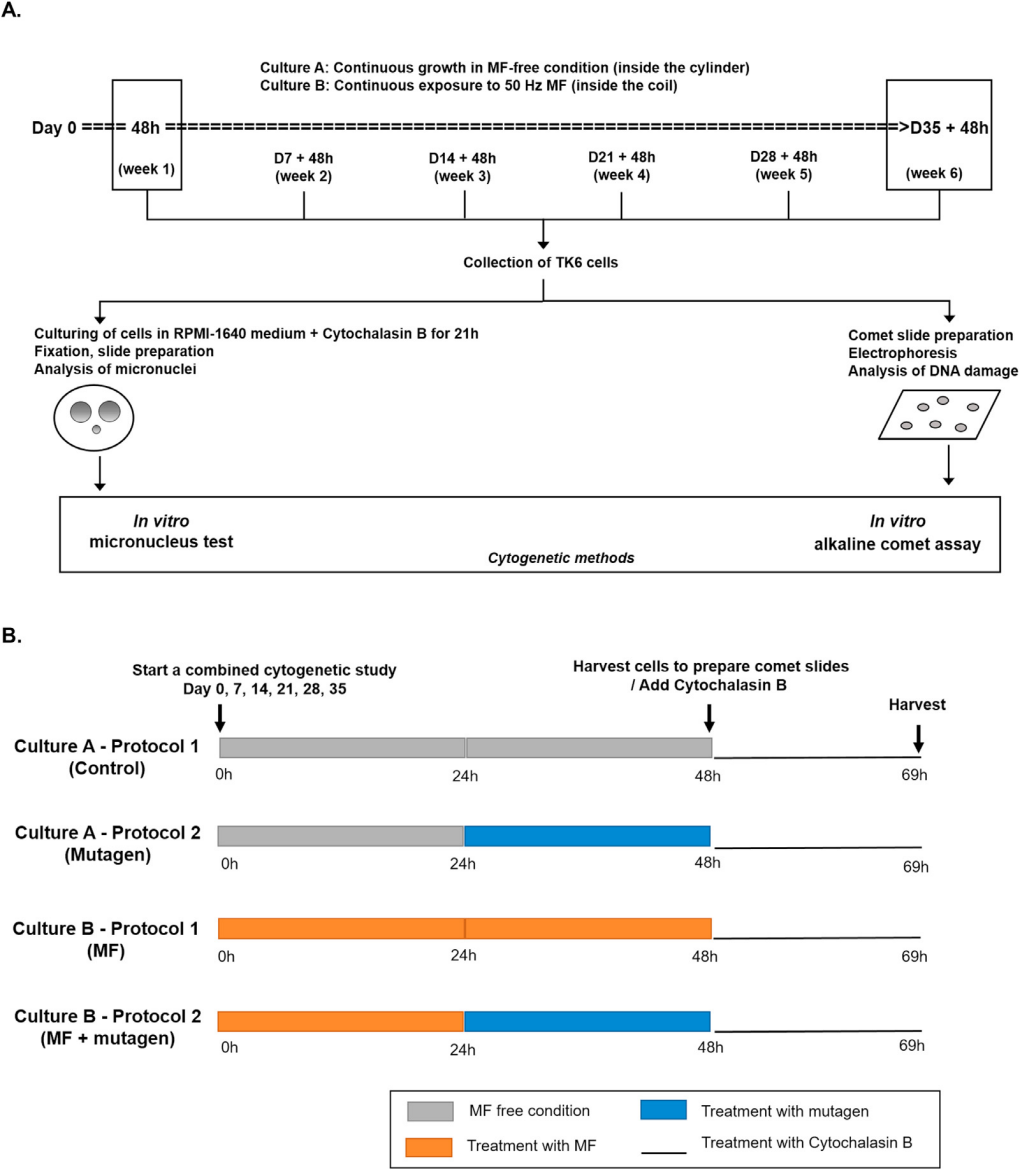
A. Schematic representation of the protocol for a combined *in vitro* cytogenetic study to investigate the effects of long-term exposure to extremely low-frequency magnetic fields (ELF-MF) in TK6 cells (protocol 1). By extending the protocol presented in A with additional treatment conditions, the impact of long-term pre-exposure to ELF-MF on the sensitivity of cells to the damage induced by known chemical mutagens can be simultaneously assessed. The details of the four different treatment conditions on TK6 cells for such an extended study are presented in B. Briefly, cells collected from Culture A and B on day 0, 7, 14, 21, 28, 35 are exposed after 24 h to a known chemical mutagen for 24 h (protocol 2).

´Long-term´ exposure implies that cells are continuously and repeatedly exposed to test agents over a long period of time (during multiple cell divisions). To this extent, two cultures (Culture A and B) of TK6 cells are maintained for at least six consecutive weeks. Culture A (no MF exposure) is put inside a mu-metal cylinder which protects cells from unintentional ELF-MF exposure (see ‘Precautions related to ELF-MF exposure system and test environment’). Culture B (continuous MF exposure) is placed inside a coil (cylindrical exposure unit) [12], allowing the continuous exposure of cells to 50 Hz MF at different flux densities. At the start of each week (Day 0, 7, 14, 21, 28, and 35), part of the cells from both cultures are collected to analyse genetic damage with the COM and MN (Fig. 1A).

In order to simultaneously investigate the effects of long-term MF exposure on TK6 cells and the sensitivity of MF long-term pre-exposed cells to known chemical mutagens, additional experimental conditions can be included (Fig. 1B). In this extended experimental set-up, there are in total four treatment conditions, namely

• Control (Culture A - Protocol 1): unexposed TK6 cells;
• Mutagen (Culture A - Protocol 2): TK6 cells exposed for 24 h to the known chemical mutagen. Exposure to mutagen starts at 24 h after the collection of the cells at day 0, 7, 14, 21, 28, or 35;
• MF (Culture B - Protocol 1): TK6 cells long-term exposed to 50 Hz MF;
• MF + mutagen (Culture B - Protocol 2): TK6 cells pre-exposed for long-term to 50 Hz MF followed by exposure to the known mutagen for 24 h. Exposure to mutagen starts at 24 h after the collection of the cells at day 0, 7, 14, 21, 28, or 35.

The known chemical mutagens to which TK6 cells are exposed in the COM and MN are ethyl methanesulfonate (EMS; 0.25 mM) and methyl methanesulfonate (MMS; 2 µg/mL), respectively. The concentrations of the mutagens were selected based on the results of tests with a wide range of mutagen concentrations (Supplementary Fig. 1; Supplementary Table 1).

The effects of long-term MF exposure on TK6 cells are assessed by comparing the results of MF cells with those of the control cells. To evaluate the impact of MF pre-exposure on the sensitivity of cells to known chemical mutagens, results of (MF + mutagen) cells are compared with those of the mutagen cells whereas the unexposed cells serve as the negative control.

### Required reagents and equipment

#### Cells

Experiments are performed in TK6 cells, a well-studied cell line that is recommended by the Organisation for Economic Co-operation and Development [18] for the *in vitro* MN. Like other *in vitro* cultured cell lines, TK6 cells have several advantages such as a homogeneous genetic background, easy storage and reduced cell variability [3], but in addition to that, they also have a high proliferation rate and produce fewer misleading positive results due to p53-competence [22]. Furthermore, the cells are non-adherent and therefore easier to maintain as trypsinization is not needed. The frozen stocks of TK6 (ECACC 95111735) are stored in liquid nitrogen.

#### Reagents

• Absolute ethanol (VWR)
• Acridine Orange (VWR)
• Cytochalasin B (CytB) (Drechslera dematioidea, Sigma-Aldrich)
• Dimethyl sulfoxide (DMSO) (276855, Sigma-Aldrich)
• Denaturated ethanol (VWR)
• Denaturation/electrophoresis buffer (pH>13): NaOH 300 mM, EDTA 1.27 mM (VWR)
• Ethyl methanesulfonate (EMS) (Sigma-Aldrich)
• Fixative solutions: Fixative 1 (methanol:glacial acetic acid with a ratio of 3:1); Fixative 2 (methanol: glacial acetic acid with a ratio of 17:3)) (VWR)
• GelRed (3X) (VWR)
• Methyl methanesulfonate (MMS) (Sigma-Aldrich)
• Neutralisation buffer (pH 7.5): TRIS-base 0.5M (VWR)
• Low melting point Agarose (LMP) 0.8% (Thermo Fisher Scientific)
• Lysing solution: 10% DMSO, 1% Triton X-100 (Sigma-Aldrich), NaCl 2.5 M, EDTA 100 mM (Sigma Aldrich), TRIS 10 mM (VWR), and NaOH pellets (added freely until the solution reaches pH 10)
• Normal melting point Agarose (NMP) 1% (Thermo Fisher Scientific)
• Potassium chloride 0.075 M (VWR)
• RPMI 1640 culture medium with 25 mM Hepes supplemented with 10% (v/v) Fetal bovine serum, 1% Gentamycin, Glutamax, Sodium Pyruvate & Non Essential Amino Acids and 0.1% Amphoterecin B (Thermo Fisher Scientific)
• Sorensen buffer, pH 6.8 (VWR)
• Vectashield mounting medium (VWR)
• Vectashield mounting medium with 4′, 6’-diamidino-2-phenylindole (DAPI) (VWR)

#### Equipment

• Exposure systems (Coil configuration was used to generate 50 Hz MF at different flux densities. The detailed description of the exposure system is presented in the second part of this manuscript)
• Mu-metal cylinder (Meca Magnetic) (This cylinder can shield the control cells against background ELF-MFs. Its detailed description and validation are presented in the second part of this manuscript)
• Incubator at 37 °C, 5% CO_2_ (Binder incubator, VWR)
• Biological safety cabinet (class II; BioVanguard Green Line, Telstar)
• Chemical hood
• Water bath at 37 °C (Sub Aqua Pro, Grant)
• Benchtop centrifuge (Heraeus, Multifuge 1S, ThermoFisher Scientific)
• Heating block at 36 °C (QBD2, Grant)
• Electrophoresis chamber with power supply (COMET-40 system, SCIE-PLAS, LTD)
• pH meter (pH7110, Inolab)
• Fluorescent microscope (AxioImager.Z2) supplied with a camera and connected to the Automated Scanning System Metafer4 (Metasystems)

### Detailed experimental procedure

#### Cell culturing

• Prepare 2 different cultures (Culture A and B) of TK6 cells. Put cells of Culture A inside a mu-metal cylinder and cells of culture B in the coil generating 50 Hz MF. Maintain the cells for 6 weeks at 37 °C in a 5% CO_2_ humidified atmosphere at a cell concentration ranging between 2 × 10^5^ and 1 × 10^6^ cells/mL.

### *In vitro* alkaline comet assay

*General notes*

• Super-frosted slides precoated with 1% NMP need to be prepared at least three months in advance.
• After collection of the cells, perform all steps in a dark room (without direct sunlight, but yellow light without UV) to protect samples from extra DNA damage.
• Before preparing comet slides, put 600 µL of LMP 0.8% into Eppendorf's tubes in heating block at 36 °C.
• Lysing working solution should be prepared fresh and kept at 4 °C.
• Denaturation/ electrophoresis buffer need to be freshly prepared and the temperature should be ± 15 -17 °C.

*Step 1: cell seeding*

• At day 0, 7, 14, 21, 28 and 35, cultivate TK6 cells of culture A and B in two different 6-well plates at a concentration of 50000 cells/mL (4 mL cell suspension per well). In order to have replicates for each treatment condition, cells are seeded in 6 wells for each culture.
• Incubate cells for 24 h at 37 °C, 5% CO_2_. Put cells of Culture A inside a mu-metal cylinder and cells of culture B in the coil.

*Step 2: cell exposure*

• Protocol 1: Add 100 µL of RPMI 1640 fresh medium to three wells from both Culture A and B. Put cells of Culture A inside a mu-metal cylinder and cells of culture B in the coil.
• Protocol 2: Add 100 µL of EMS 10 mM into three wells from each culture. Put both cells of Culture A and B inside a mu-metal cylinder.
• Incubate for 24 h at 37°C, 5% CO_2_.

Note: After 24 h of cultivation, the cell concentration in each well should be around 114000–200000 cells/mL, which is equivalent to 1.2–2 cell cycles.

Step 3: cell collection and comet slide preparation

• Transfer the contents of each well into 15 mL round-bottom tubes for centrifugation for 5 min at 102 x g.
• Discard the supernatant, resuspend cell pellets with 1 mL of new medium and put on ice to prevent further DNA damage.
• Mix 50 µL of cell suspension from each culture with 600 µL LMP (36°C) and avoid air bubbles.
• Load 75 µL cell/LMP mixture onto pre-coated slides and immediately cover with a 24 × 24 mm coverslip.
• Keep slides for 5 min on an ice cold plate in the fridge and then leave at room temperature for 2 min.
• Remove the coverslips and randomly distribute slides in a jar with ice cold lysing buffer and keep for at least an hour in the fridge.

*Step 4: cell denaturation - electrophoresis - neutralisation*

• After lysis, keep cells in denaturation/electrophoresis buffer pH>13 in the electrophoresis chamber for 40 min to allow DNA unwinding.
• To start electrophoresis, connect the electrophoresis chamber to the power supply with the voltage set at 1 V/cm for 20 min.

Note: It is important to always perform electrophoresis under the same conditions (voltage of 1 V/cm, same amount of buffer, which should be enough to cover the slides completely, and same temperature). For a homogenous distribution of buffer, a circulating pump should be connected to the electrophoresis chamber (low speed). This process is preferably performed at 4 °C. If there is no cooling system connected to the electrophoresis chamber, ice can be put around the electrophoresis chamber as an alternative option.

• As soon as the electrophoresis is finished, remove all slides from the electrophoresis chamber and rinse them three times with neutralization TRIS buffer pH 7.5 for 5 min.
• Put slides into ice-cold absolute ethanol for 10 min at 4 °C.
• Air-dry slides overnight in the dark at room temperature.

*Step 5: staining - scoring of DNA damage - statistical analysis*

• Rehydrate slides by adding 200 µL distilled H_2_O on each slide and put 24 × 50 mm coverslip on for 10 min.
• Remove coverslip, add 100 µL GelRed 3x to each slide and put coverslips for the next 10 min.
• Remove coverslip and wash the excess of GelRed by rinsing slides with water.
• To avoid GelRed from fading and gel drying out, put 2 drops of Vectashield mounting medium onto each slide.
• Keep slides for at least 15 min in the fridge (in a box filled with humidified paper) before analysis.
• Analyze the percentage of DNA in the tail of the comet.

Analysis can be done automatically with the fluorescent microscope (AxioImager.Z2) supplied with a camera and connected to comet imaging software (Automated Scanning System Metafer4 – CometScan = Automated system for unattended detection and analysis of Comet assay slides, based on the scanning platform Metafer4). A predefined area on each gel is scanned for the presence of single cells with parameters that correspond to those of the settings of a chosen comet assay classifier (program). Images of 200 matching cells are captured and the percentage of DNA in the comet tail for each cell is calculated automatically. Results of percentage of DNA in the comet tail are displayed for each cell of the sample in customizable list format and they are saved together with all scan information and the gallery of all captured images in an individual file for each sample. After automated scoring, cell selection is done to eliminate the incorrect images (Supplementary Fig. 2). The percentage of DNA damage is determined as the median value of that percentage of the first 50 detected cells (x2) in each treatment condition. For the statistical analysis, the Mann-Whitney U-test is used. This test does not require normal distribution data to compare variables between two independent groups. It evaluates if a randomly selected value from one group (for example the exposed cells) is significantly different from a randomly selected value from another group (for example unexposed cells).

### *In vitro* micronucleus test

General notes

• Clean MN slides in advance by keeping slides in acetic acid 10% solution for at least 24 h, followed by immersion in denatured ethanol for 24 h. Afterwards, dry slides using medical wipes.
• Perform the fixation steps in a chemical hood.
• Prepare the hypotonic solution and the fixative solutions fresh and keep at 35 °C or 4 °C, respectively.
• Always perform centrifugation at 102 x g for 5 min.

*Step 1 & 2: cell seedling and exposure*

• Perform both steps as described for the *in vitro* alkaline comet assay but use MMS (2 µg/mL) instead of EMS. In order to have the final concentration of 2 µg/mL MMS, add 80 µL of MMS 100 µg/mL into three wells from each culture.

*Step 3: treatment with CytB*

• Transfer the contents of each well into 15 mL round-bottom sterile tubes and centrifuge.
• Discard the supernatant and resuspend the cell pellets with 5 mL of new medium supplemented with CytB 3 µg/mL.
• Incubate the cells for 21 h in 6-well plates. Put cells of both cultures in the mu-metal cylinder.
• After 21 h, transfer cells to 15 mL centrifuge tubes for the fixation step.

Note: It is important to keep TK6 cells in CytB for 21 h, no longer. An experiment with cells treated with CytB for different periods showed that at this time point, the majority of cells has undergone nucleus replication (Supplement Table 2).

*Step 4: cell fixation*

• Centrifuge tubes and aspirate the supernatant, but leave enough supernatant to homogenize the cell pellet.
• Homogenize the cell pellets by vortexing, followed by a hypotonic shock with 1 mL KCl (0.075 M at 35 °C). Add the KCl slowly (drop by drop) into each test tube while gently vortexing. Leave the tubes for exact 10 min at room temperature.
• Pre-fix cells by slowly adding 1 mL of (freshly prepared) ice-cold fixative 1 into each tube in the same order and same manner as for the hypotonic solution.

Note: The pre-fixation step is very important to preserve the cell cytoplasm. Without pre-fixation, most of the cytoplasm of the TK6 cells will be lost.

• Leave cells with fixative 1 at room temperature for 10 min.
• Centrifuge and aspirate supernatant but leave enough supernatant to homogenize the cell pellet.
• For cell fixation, add dropwise 1 mL ice-cold fixative 1 into each tube while gently vortexing, followed by the addition of 4 mL of ice-cold fixative 1.
• Gently vortex tubes and centrifuge.
• Aspirate the supernatant, but leave enough to homogenize the cell pellet.
• Repeat the three prior steps, but using fixative 2, instead of fixative 1.

Note: Combining fixative 1 and fixative 2 allows the cells become round and big enough for analysis without cytoplasm loss (Supplementary Fig. 3).

• To prepare micronucleus slides, add 2-3 drops of the cell suspension on pre-cleaned slides and make sure cells are equally spread throughout the slide. Prepare 3 slides for each condition.
• Dry slides overnight.

*Step 5: DAPI staining – scoring of micronuclei – statistical analysis*

• Add 2 drops of Vectashield mounting medium containing DAPI on each slide.
• Put 24 × 50 mm coverslip onto each slide.
• Leave slides at room temperature for at least 15 min before starting microscopic analysis.
• Analyse the number of micronuclei.

Analysis of each slide can be done automatically using a fluorescent microscope supplied with a camera and connected to the Metafer4 software. For the micronucleus test, a classifier with preset parameters to automatically detect bi-nucleated cells with MN is used. Number of binucleated cells is set to 2000 per slide. After automated analysis, the detected micronuclei are analysed visually (choose manually Objective 40x and DAPI filter on the microscope) in order to eliminate false positive results and evaluate whether they fulfil the criteria described in OECD 487 test guideline: *In vitro* mammalian cell micronucleus tests. For each condition, two slides are scanned and the average result is used. The results are described as the number of micronuclei per 2000 cells.

For the statistical analysis, Fisher's Exact test is performed to evaluate whether there is a statistically significant increase in micronuclei in one treatment condition compared to another.

*Step 6: Assessment of cytotoxicity*

• Stain cells for 1 min in Sörensen buffer with 0.1% acridine orange in a staining jar.
• Wash slides 3 times for 3 min in Sörensen buffer (pH 6.8) in staining jars.
• Before analysis, put 200 µL Sörensen buffer on each slide.
• Determine the Cytokinesis-Block Proliferation Index (CBPI) and calculate cytostasis.

CBPI (1) serves as an indicator for the effects of the test agent on cell proliferation. The CBPI values can also be used to assess cytotoxicity based on the % cytostasis (equation (2)). A CBPI of 1 (all cells are mononucleate) is equivalent to 100 % cytostasis.

(1) CBPI=(M1+2xM2+3xMm)/N.

Where: M1, M2, Mm represent the number of respectively mononucleated, binucleated and multinucleated cells. N is the number of cell scored (=500)

(2) $$\text{Cytostasis}\;\%=100-100\left[\left(\text{CBP}I_{\text{EP}}-1\right)/\left(\text{CBP}I_{\text{NC}}-1\right)\right]$$

CBPI_EP:_ Cytokinesis-block proliferation index of treated cells

CBPI_NC:_ Cytokinesis-block proliferation index of unexposed cells

## Precautions related to ELF-MF exposure system and test environment

### Exposure system

#### Characteristics of the exposure system

The exposure system was designed and built at the Department of Applied Electricity of the University of Liège. The system has previously been described in Maes et al. [12] and was already repeatedly used in studies performed by members of the BioElectroMagnetics Group ([25]; e.g. [12,14]). Briefly, a cylindrical exposure unit (380 turn coil, 42 cm long, 20 cm inner diameter) placed inside an incubator at 37°C is used to generate 50 Hz MF. The dimensions of the coil were chosen so as to obtain a sufficiently large zone in which the cell cultures can be exposed to a nearly constant magnetic field. A main unit supplies the 50 Hz current flowing in the coil using a variable voltage autotransformer. Insertion of series impedances allows selecting different ranges from 0 up to about 1800 µT.

#### Magnetic field validation

The validation of the amplitude of 50 Hz MF inside the coil was done by the Applied and Computational Electromagnetics unit of ULiège using a field probe (Maschek: ESM-100 S.N. 971739) with the following characteristics:

• Range of magnetic induction measurement (B): 1 nT – 20 mT
• Bandwidth: 5 Hz – 400 kHz (with filters at 50 Hz), 16 Hz 2/3, 5 Hz-400 kHz, 5 Hz-2 kHz, 2 kHz-400 kHz.
• Accuracy: ± 5%.

Magnetic inductions at 1000 µT, 500 µT, 100 µT, 50 µT and 10 µT were validated. To obtain the desired uniform magnetic induction, the culture plate has to be positioned in the middle of the coil both vertically and longitudinally. Due to its power supply mode, the magnetic induction contains harmonics (i.e. a frequency of 50 Hz plus components that are multiples of 50 Hz).

#### Temperature validation

The variation in temperature was studied to check whether current flowing in the coil could induce significant thermal effects on tested cells. When the coil was put inside the incubator, different magnetic flux densities including 50 µT, 100 µT, 500 µT were applied and the temperature inside and outside the coil was recorded (Table 1). At 500 µT, there was a slight increase in the recorded temperature inside the coil. Outside the coil where the control cells are usually kept, no significant change in temperature was observed in all test conditions.

**Table 1:** Temperature monitoring inside and outside the exposure system for different magnetic fields. Temperature was measured using hand held digital thermometer (VWR).

Registered magnetic field (µT)	Temperature (°C)	
	Inside coil	Outside coil
0	37.0 ± 0.0	37.0 ± 0.0
50	37.0 ± 0.0	37.0 ± 0.0
100	37.0 ± 0.0	37.0 ± 0.0
500	37.2 ± 0.2	37.0 ± 0.0

### Test environment

#### Test conditions

Measurements with an Emdex II magnetic field meter were made to ensure that the conditions of the magnetic environment are appropriate. The following parameters were applied:

• Sampling rate: 3 s
• Recording of the resultant fields in broadband (40-800 Hz) and harmonics (100-800 Hz).

**Fig. 2:**
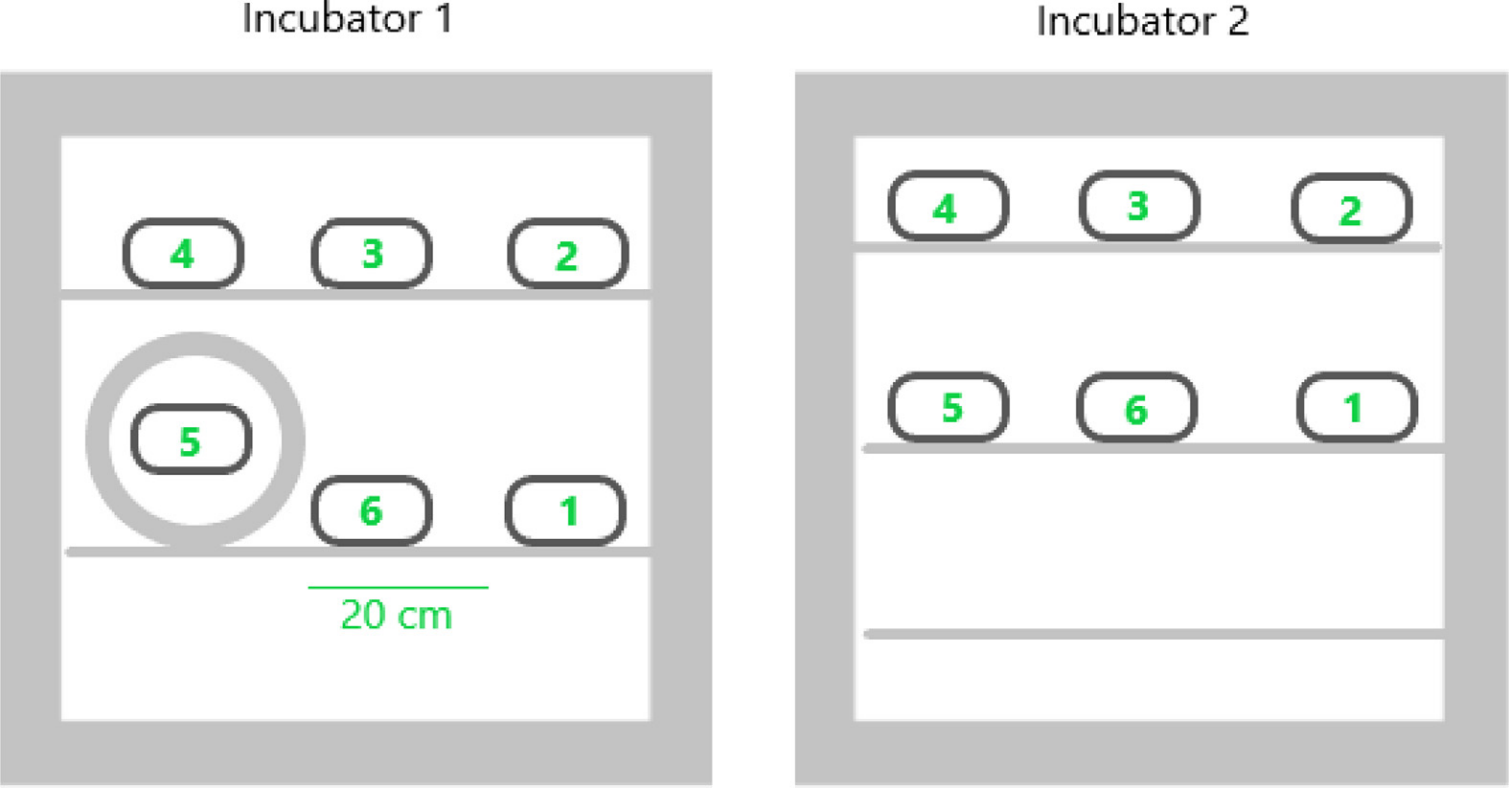
Positions of the Emdex II to measure MF in both incubators (circle: view of coil setup). In each position, measurements were done at three different points: front (close to the door of the incubator), middle, and back (close to the back of the incubator).

The device was positioned in the incubator as illustrated in Fig. 2.

### Measurement of background MF inside incubator

**Fig. 3:**
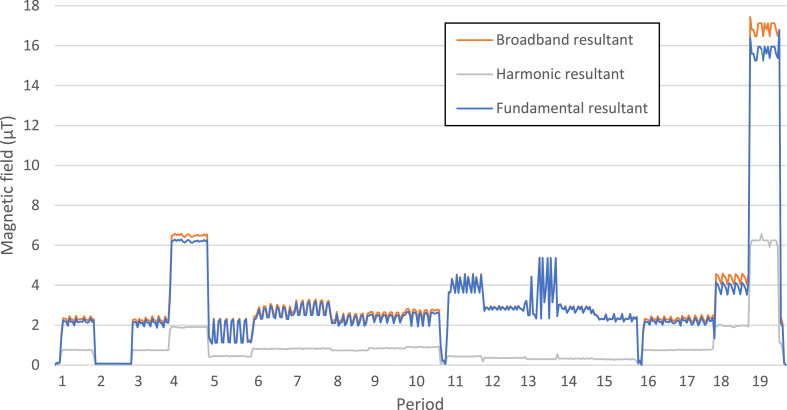
Results of MF measurements under different conditions in two different incubators. *Measurement conditions in incubator 1 (Binder incubator)* Period 1: In incubator 1 on – Position 1 / Middle - Coil off; Period 2: In incubator 1 off – Position 1 / Middle - Coil off; Period 3: In incubator 1 on – Position 1 / Middle - Coil off; Period 4: In incubator 1 on – Position 1 / Back - Coil off; Period 5: In incubator 1 on – Position 1 / Front - Coil off; Period 6: In incubator 1 on – Position 2 / Middle - Coil off; Period 7: In incubator 1 on – Position 3 / Middle - Coil off; Period 8: In incubator 1 on – Position 4 / Middle - Coil off; Period 9: In incubator 1 on – Position 5 / Middle - Coil off; Period 10: In incubator 1 on – Position 6 / Middle - Coil off;*Measurement conditions in incubator 2 (Thermo incubator)* Period 11: In incubator 2 on – Position 1 / Middle - Coil off; Period 12: In incubator 2 on – Position 1 / Back - Coil off; Period 13: In incubator 2 on – Position 1 / Front - Coil off; Period 14: In incubator 2 on – Position 2 / Middle - Coil off; Period 15: In incubator 2 on – Position 4 / Middle - Coil off; *Measurement conditions in incubator 1 (Binder incubator) in Position 1 with different magnetic flux densities registered in the coil* Period 16: In incubator 1 on – Position 1 / Middle - Coil off; Period 17: In incubator 1 on – Position 1 / Middle - Coil 10 µT; Period 18: In incubator 1 on – Position 1 / Middle - Coil 100 µT; Period 19: In incubator 1 on – Position 1 / Middle - Coil 500 µT.

No electrical devices or wiring were placed in the surroundings of the incubator. The measurements confirmed the absence of magnetic fields coming from outside the incubator (Fig. 3, Period 2). The multiple recordings at position 1 / middle in incubator 1 (coil off) (Fig. 3, Periods 1, 3 and 16) also demonstrate that measurements at different time points are reproducible.

However, the measurements in both incubators revealed non-negligible background levels of ELF-MFs (Fig. 3) from the incubator itself. In addition, these background fields varied between the incubators. In incubator 1, when the coil was off, the background fields ranged from 2.2 µT to 6.2 µT depending on the position of the measuring device (Fig. 3, Periods 3-10). In incubator 2, those values were between 2.3 µT and 5.3 µT (Fig. 3, Periods 11-15). Controls in a third incubator revealed even more differences in the background MF, with values close to 17 µT in the backend of the incubator and 3 µT in the frontend (results not shown). Among the different measurements, position 1 (middle) of the incubator 1 (Fig. 3, Period 5) showed the lowest mean MF value, but significant amplitude variations.

### Measurement of 50 Hz MF outside the energized coil

When the coil generates a field of 10, 100 and 500 µT, unintentional fields of respectively 2.2 µT, 4 µT and 17 µT were recorded in position 1, 20 cm from the coil (Fig. 3, Periods 17-19).

Measurements confirmed that unintentional 50 Hz MF exposure of cells in the incubator can be caused by different sources, namely the exposure system, and the background MF of the incubator. The control cells might thus be exposed to higher MF than those reported [9,^16^,20]. Though the level of background MF in the incubator (2.2 µT in incubator 1, but around 4 µT in incubator 2 and even more in the other incubator tested) is lower than the experimental MF levels (10 to 500 µT), this value should not be neglected, especially when performing low-intensity tests (below 10 µT) where the orders of magnitude are close. Moreover, it is worth noting that these background levels are already higher than the usual residential exposure level of the general public, which is lower than 0.1 µT [15]. Besides, epidemiological studies repeatedly found that an average daily exposure to ELF-MF of 0.4 µT or higher was associated with an approximately two-fold risk increase in childhood leukemia [9]. Therefore, in a biological experiment designated to investigate the effect of MF exposure, it is important to include a good negative control which has the exposure level close to 0 µT to observe the effect of MF exposure as well as to avoid bias between repeated tests, especially when investigating the effects of very low exposure levels.

Portelli et al. [19] hypothesized that the inhomogeneity of the background MF in incubators is a potential confounding source underlying the variability and lack of reproducibility of studies performed on cell cultures. When studying the effects of 50 Hz MF exposure in a lab, it is important to take precautions in order to avoid interference of unintentional MF from the surrounding environment. For example, when the coil generates an ELF-MF of 500 µT, the control cells are exposed to levels of about 17 µT (directly by the energized coil) (Fig. 3, Periods 19), which is higher than one of the test levels (10 µT). Use of another incubator for the control cells to circumvent this problem is not an appropriate solution as conditions in terms of background MF, temperature, CO_2_ and vibrations should be the same for the control and the exposed cells [20]. Consequently, efficient shielding of control cells against unintentional MF is a key factor in this type of studies.

### Shielding system

#### Characteristics of the shielding system

**Fig. 4:**
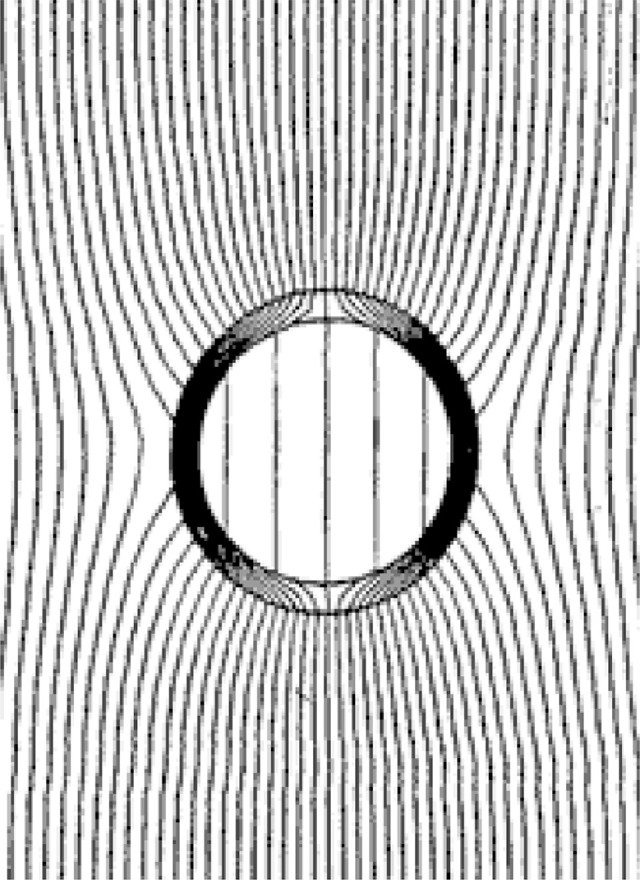
Attenuation of magnetic field by magnetostatic shielding (low-conductivity high-permeability material) (source: BBEMG website)

Mu-metal is a nickel-iron soft ferromagnetic alloy with very high permeability, which is used for shielding sensitive electronic equipment against low-frequency MF. In addition to the shielding effect of the Eddy currents, which is weak at low frequency, this kind of material offers additional shielding properties through its capacity to concentrate magnetic flux lines (Fig. 4). The use of mu-metal to protect the cells from EMF exposure was mentioned in several previous studies [5,^7^,8].

**Fig. 5:**
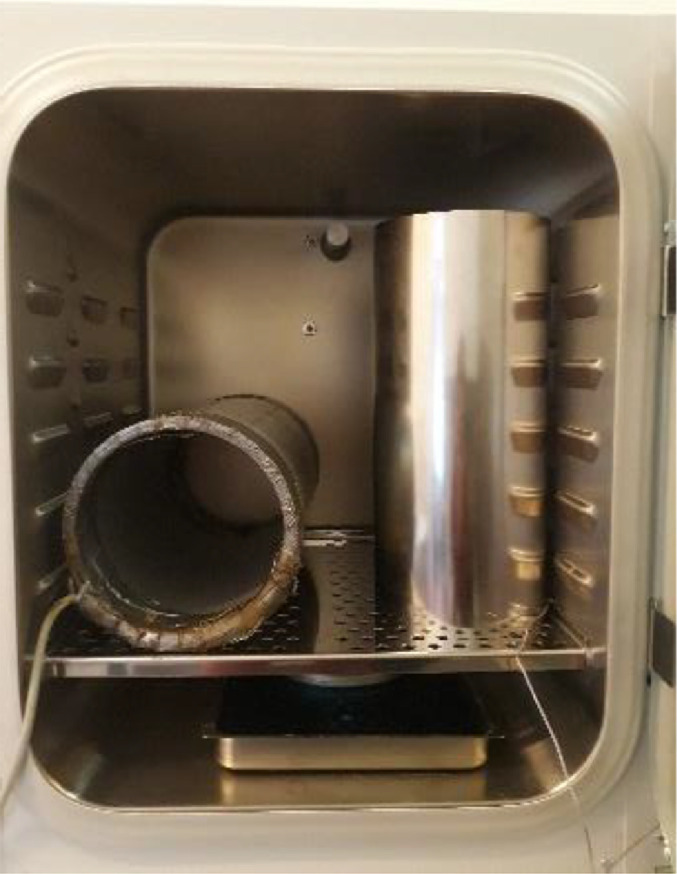
Experimental setup to study long-term exposure to ELF-MFs (the exposure system on the left side and mu-metal cylinder on the right side)

To avoid bias linked to the exposure environment and the characteristics of the incubator (temperature, CO_2_ level, humidity, vibrations,…), it is advised to place control cells in the same incubator during the experiment. Consequently, an efficient shielding is required to protect these control cells against unintentional MF originating not only from electronic circuits of the incubator, but also from the coil. In addition, temperature and air circulation should also be maintained. All these factors complicate geometry-based configuration design. After testing several configurations, a vertical cylinder proved to be the most appropriate shape to reduce MF while maintaining adequate air flow and temperature control (Fig. 5).

**Fig. 6:**
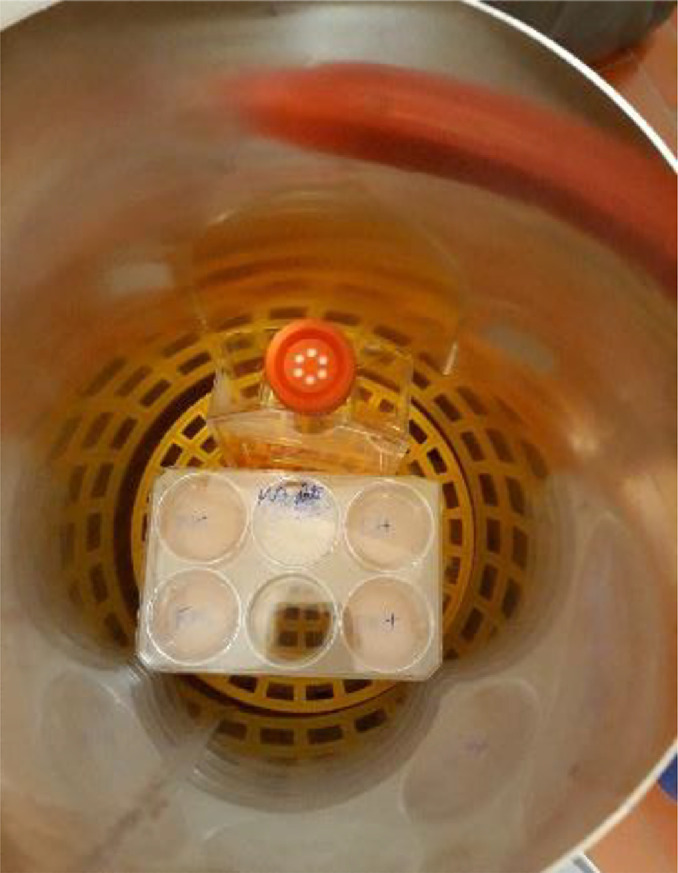
View inside the mu-metal cylinder with the support (orange holder) to position the cells at the center of the cylinder

**Fig. 7:**
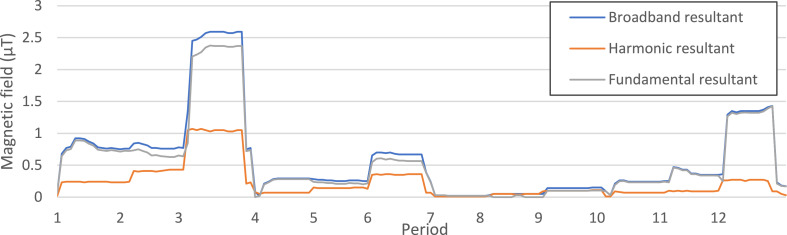
MF measurements inside the mu-metal cylinder with the coil energized at different levels. *Emdex II sensors at the bottom of the cylinder* Period 1: In incubator 1 on – Inside the cylinder - Coil off; Period 2: In incubator 1 on – Inside the cylinder - Coil 100 µT; Period 3: In incubator 1 on – Inside the cylinder - Coil 500 µT; *Emdex II sensors at 10 cm high* Period 4: In incubator 1 on – Inside the cylinder - Coil off; Period 5: In incubator 1 on – Inside the cylinder - Coil 100 µT; Period 6: In incubator 1 on – Inside the cylinder - Coil 500 µT; *Emdex II sensors at 20 cm high (middle of the cylinder)* Period 7: In incubator 1 on – Inside the cylinder - Coil off; Period 8: In incubator 1 on – Inside the cylinder - Coil 100 µT; Period 9: In incubator 1 on – Inside the cylinder - Coil 500 µT; *Emdex II sensors at 30 cm high* Period 10: In incubator 1 on – Inside the cylinder - Coil off; Period 11: In incubator 1 on – Inside the cylinder - Coil 100 µT; Period 12: In incubator 1 on – Inside the cylinder - Coil 500 µT.

The cylinder in mu-metal (2.23 kg, 40 cm long, 18 cm diameter), designed by Meca Magnetic (France), is put in an upright position with two ends open to allow the circulation of CO_2_ and constant temperature inside the cylinder. The cylinder has been thermally treated by the manufacturer (3 h at 1080 °C) in order to restore the alloy metallurgical structure to obtain optimal magnetic properties. It is used with a support (Fig. 6) to position the cells at the middle of the cylinder in which the MF is the lowest (Fig. 7).

#### Magnetic field validation

Measurements were made using the Emdex II MF meter to ensure that the levels of magnetic fields inside the cylinder are appropriate. The device was positioned in the cylinder at different heights and measurements of MF were done when the coil was energized at different levels (Fig. 7).

Based on the results, culture plates have to be located in the middle of the cylinder to have the lowest levels of MF (Fig. 7). Indeed, the graph shows that all measured values were lower than 0.1 µT, except at 500 µT. Although the MF value was higher at 500 µT (0.14 µT), the unintentional MF radiation was reduced by a factor 100 compared to the initial situation (Fig. 3, Period 19) and is considered as appropriate for the experiments.

#### Validation of cell proliferation rate

To assess the effect of the cylinder on the proliferation of the TK6 cells, two sets of cell cultures with a starting concentration of 20000 cells/mL were maintained inside and outside the cylinder. After three days, the number of cells in each culture was counted and the population doubling time (PDT) was calculated based on the following formula:

$$\text{PDT}=T\;\text{ln}\;2/\text{ln}\left(X_{T}/X_{0}\right)$$

Where T is the incubation time (hour)

X_0_ is the cell number at the beginning of the incubation time.

X_T_ is the cell number at the end of the incubation time.

Comparison of PDTs of cells inside and outside the cylinder shows that the cylinder had no effect on cell proliferation (Table 2). Also, the temperature was the same inside and outside the cylinder during the tests (results not shown). These results demonstrate that the mu-metal cylinder efficiently shields cells against background MF, without having significant impact on cell proliferation and cell culture environment. This passive shielding method can be applied in any commercial biological incubators without modifications required.

**Table 2:** PDT of TK6 cells inside and outside cylinder.

Repeat	Population doubling time	
	Inside cylinder	Outside cylinder
1	15.57	14.79
2	15.02	15.29
3	14.85	14.89
4	15.26	14.71

## Conclusion

Though long-term exposure to ELF-MFs attracts much attention, the number of experimental studies, especially *in vitro* studies in which cells are long-term exposed to MF, are sparse. To our knowledge, this is the first article that provides detailed protocols of a combined COM/MN study on TK6 cells to investigate the effects of long-term exposure to ELF-MFs as well as the combined effects of ELF-MFs and chemical mutagens. Furthermore, with this manuscript, the authors highlight the need of thorough assessment of the MF exposure system and exposure environment to ensure reproducible test results. The background MF, which always exist inside the incubator, needs to be avoided when performing tests on the effects of MF exposure, especially when investigating very low exposure levels. The solution of using mu-metal cylinder to shield the background MF is, though simple, but efficient to create the MF free environment for testing.

## Declaration of Competing Interest

The authors declare that they have no known competing financial interests or personal relationships that could have appeared to influence the work reported in this paper.

## Appendix Supplementary materials

Image, application 1
