# Supplementary material for: In vitro 50 Hz magnetic field long-term exposure: Cytogenetic tests on human lymphoblastoid TK6 cells and validation of the test environment
Source: MethodsX. 2020 Sep 26;7:101071. doi: 10.1016/j.mex.2020.101071 (PMC7558212; doi:10.1016/j.mex.2020.101071)
Supplement: Supplementary file 1 [file mmc1.docx]

**Supplementary material *and/or* Additional information:**

**Supplementary Figure 1.** Percentage of DNA damage in the comet tail after exposure of TK6 cells to various concentrations of ethyl methanesulfonate (EMS).

| 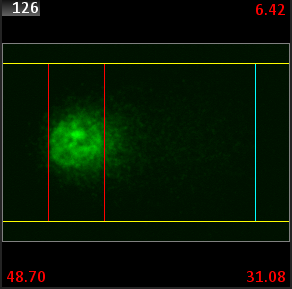 | 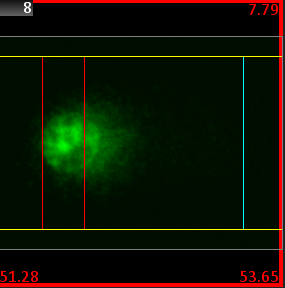 |
| --- | --- |
| A: correct comet image. | B: incorrect comet image. The software failed in distinguishing the area of head and tail of the comet, resulting in a higher percentage of DNA in the tail. |

**Supplementary Figure 2.** Pictures of acceptable comet image (A) versus incorrect comet image (B).

| 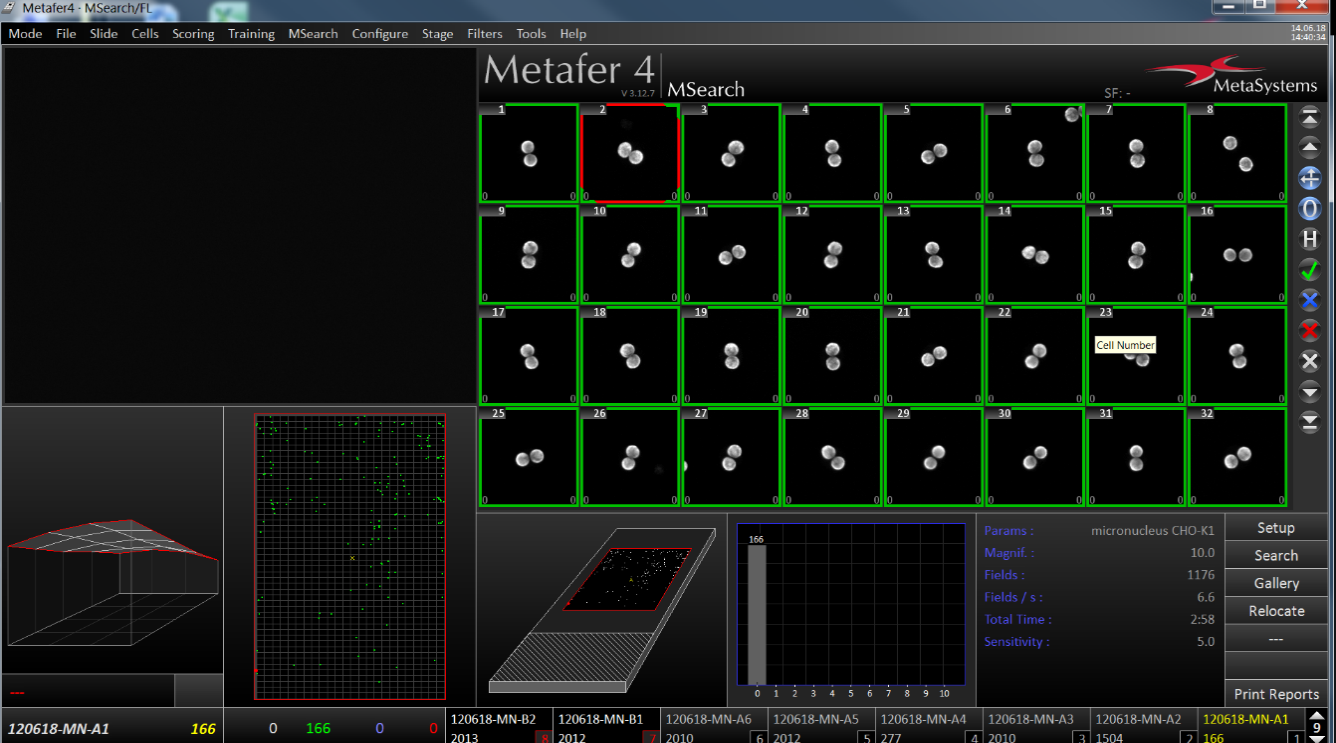 | 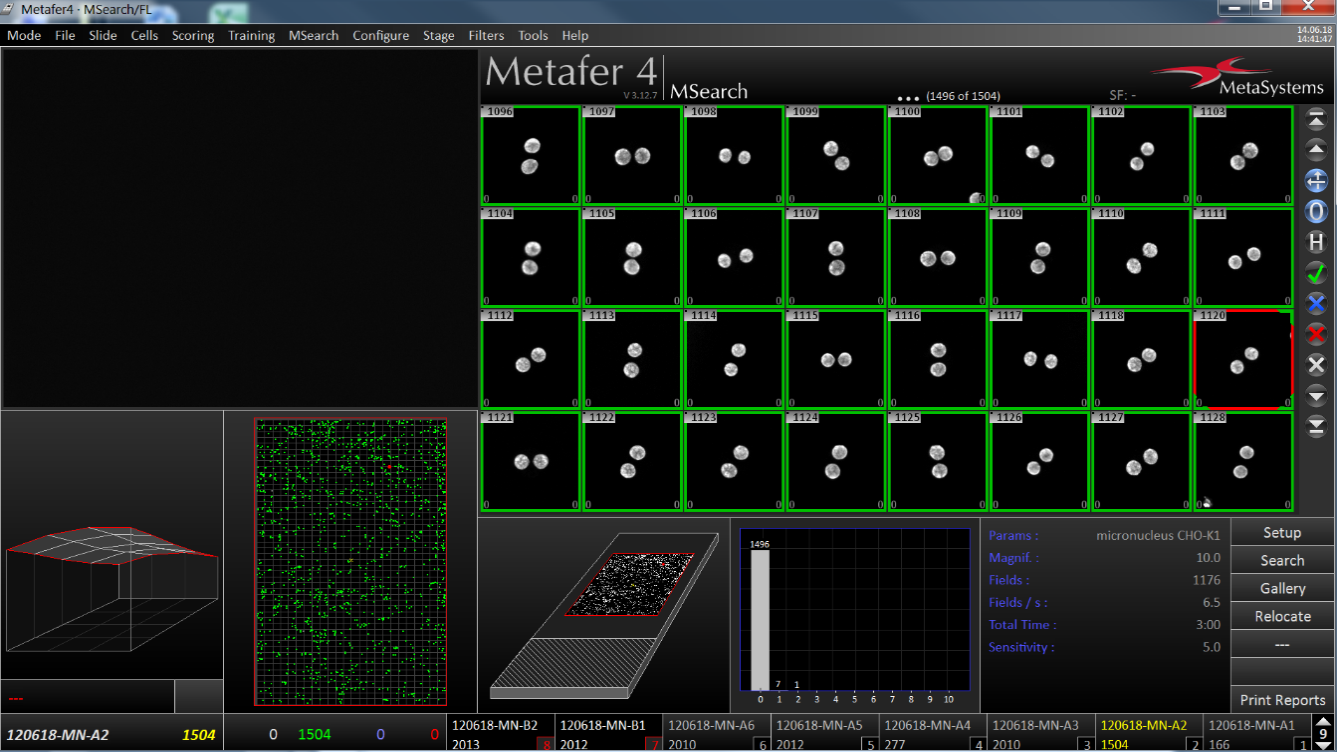 | 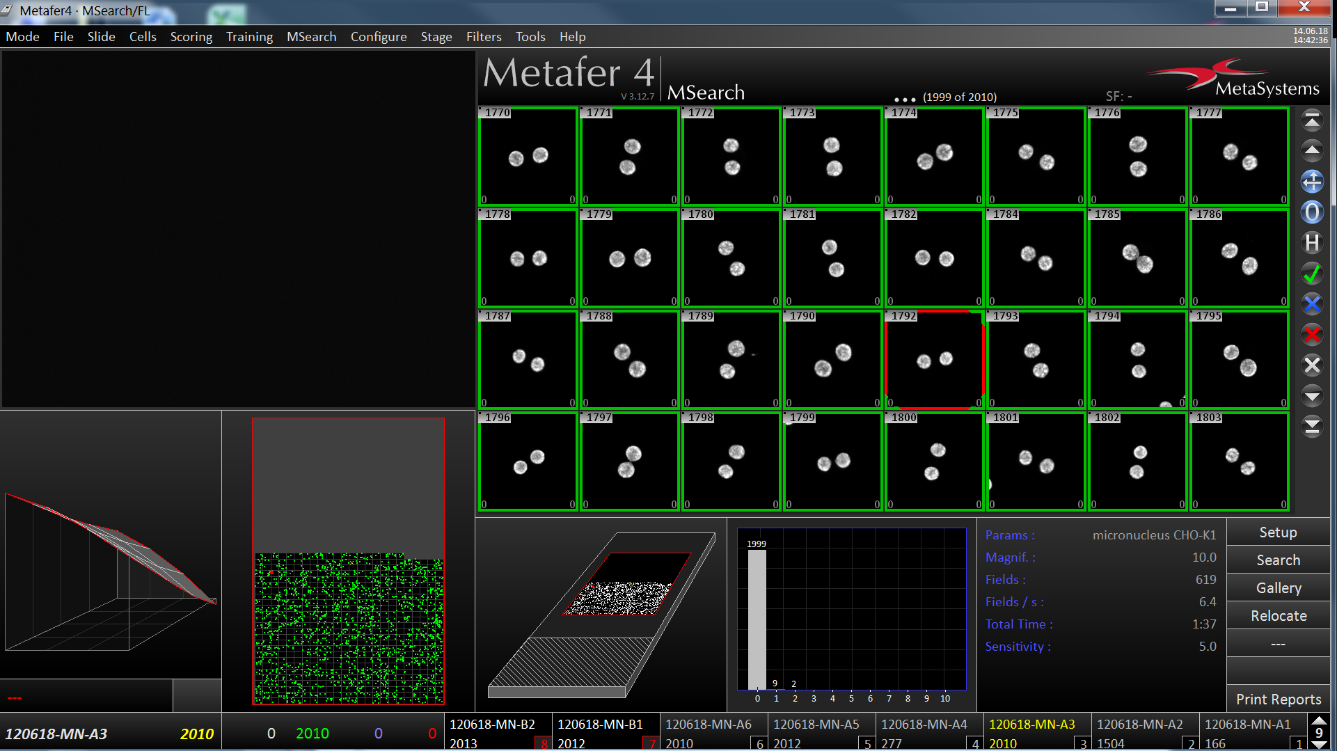 | 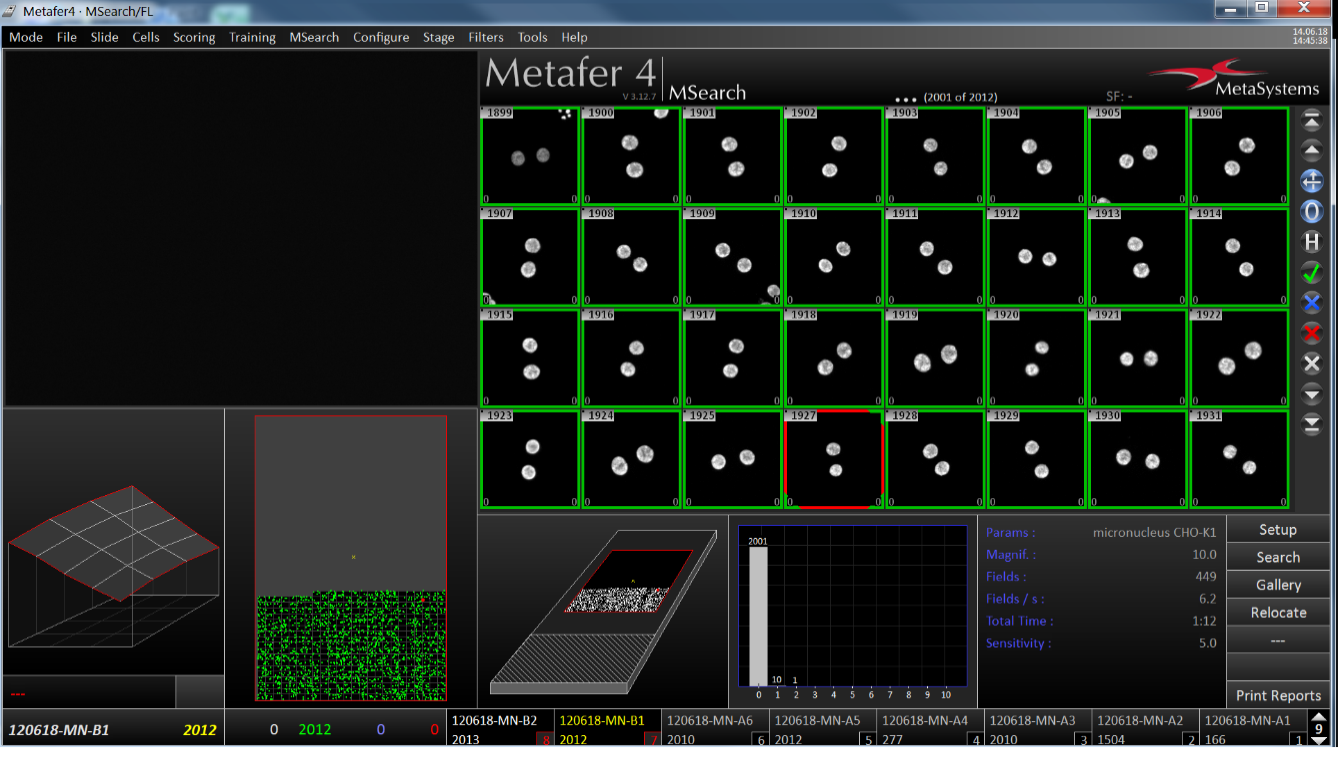 |
| --- | --- | --- | --- |
| A | B | C | D |

**Supplementary Figure 3.** Automated scanning results of micronucleus slides showing the effects of different acetic acid concentrations in fixative 2 on cell quality. A, B, C, D are results when 5%, 10%, 15%, and 25% acetic acid were used respectively. Results show that applying fixative 2 with 15% acetic acid (methanol: glacial acetic acid with a ratio of 17:3) produced high quality cells without cytoplasm loss.

**Supplementary Table 1.** Number of micronuclei per 2000 binucleated cells, CBPI value and % cytostasis in TK6 cells exposed to various concentration of methyl methanesulfonate (MMS)

| **MMS (µg/ml)** | **# MN/2000 cells** | **CBPI** | **Cytostasis (%)** |
| --- | --- | --- | --- |
| 0 | 7.83 ± 4.00 | 1.98 | 0 |
| 1.5 | 19.34 ± 0.64 | 1.92 | 6.12 |
| 2 | 49.90 ± 10.66 | 1.79 | 19.71 |
| 2.5 | 51.91 ± 12.39 | 1.73 | 25.50 |
| 5 | 148.21 ± 31.14 | 1.55 | 43.90 |
| 7.5 | 140.08 ± 26.22 | 1.61 | 37.89 |

**Supplementary Table 2**. Number of micronuclei per 2000 binucleated cells and CBPI values in TK6 cells when different treatment durations with CytB were applied

| **Time (hour) in CytB** | **# MN/2000 cells** | **# mononucleate cells** | **#binucleate cells** | **# multinucleate cells** | **CBPI** |
| --- | --- | --- | --- | --- | --- |
| 18 | 10.31 ± 2.12 | **127** | 348 | **25** | 1,80 |
| 21 | 7.83 ± 4.24 | 81 | 349 | **72** | 1,98 |
| 24 | 8.83 ± 5.66 | 60 | 325 | **116** | 2,11 |

For the *in vitro* MN test, it is recommended to treat cells with CytB during 1.5 to 2 cell cycles. For this reason, the effect of different treatment times with CytB (18, 21, and 24 hours) was investigated. The results showed that when CytB was used for 18 hours, there were 127 mononucleated cells, which is equal to 25.4% cells that have not divided. These data are comparable to those obtained with cells exposed to 2 µg/ml MMS for 24 hours, followed by 21 hours treatment with CytB. When cells were treated with CytB for 24 hours, there was a huge number of multinucleated cells, which should be avoided too. Therefore, 21 hours is the most appropriate duration to treat TK6 cells with CytB.
